# In smokers, Sonic hedgehog modulates pulmonary endothelial function through vascular endothelial growth factor

**DOI:** 10.1186/s12931-017-0590-1

**Published:** 2017-05-23

**Authors:** Priscilla Henno, Stanislas Grassin-Delyle, Emeline Belle, Marion Brollo, Emmanuel Naline, Edouard Sage, Philippe Devillier, Dominique Israël-Biet

**Affiliations:** 1 Sorbonne Paris Cité, Université Paris-Descartes, Paris, France; 2 AP-HP, Hôpital Européen Georges Pompidou, Service de Physiologie, Explorations Fonctionnelles Respiratoires et du Sommeil, 75015 Paris, France; 3 UPRES EA220, Université Versailles Saint-Quentin, Université Paris-Saclay, F-92150 Suresnes, France; 4 Plateforme de Spectrométrie de Masse & INSERM UMR1173, UFR Sciences de la Santé Simone Veil, Université Versailles Saint Quentin, Université Paris-Saclay, 78180 Montigny-le-Bretonneux, France; 5 Département des Maladies des Voies Respiratoires, Hôpital Foch, F-92150 Suresnes, France; 6 Service de Chirurgie Thoracique, Département des Maladies des Voies Respiratoires, Hôpital Foch, F-92150 Suresnes, France; 7 AP-HP; Hôpital Européen Georges Pompidou, Service de Pneumologie, 75015 Paris, France

## Abstract

**Background:**

Tobacco-induced pulmonary vascular disease is partly driven by endothelial dysfunction. The Sonic hedgehog (SHH) pathway is involved in vascular physiology. We sought to establish whether the SHH pathway has a role in pulmonary endothelial dysfunction in smokers.

**Methods:**

The ex vivo endothelium-dependent relaxation of pulmonary artery rings in response to acetylcholine (Ach) was compared in 34 current or ex-smokers and 8 never-smokers. The results were expressed as a percentage of the contraction with phenylephrine. We tested the effects of SHH inhibitors (GANT61 and cyclopamine), an SHH activator (SAG) and recombinant VEGF on the Ach-induced relaxation. The level of VEGF protein in the pulmonary artery ring was measured in an ELISA. SHH pathway gene expression was quantified in reverse transcriptase–quantitative polymerase chain reactions.

**Results:**

Ach-induced relaxation was much less intense in smokers than in never-smokers (respectively 24 ± 6% and 50 ± 7% with 10^−4^M Ach; *p* = 0.028). All SHH pathway genes were expressed in pulmonary artery rings from smokers. SHH inhibition by GANT61 reduced Ach-induced relaxation and VEGF gene expression in the pulmonary artery ring. Recombinant VEGF restored the ring’s endothelial function. VEGF gene and protein expression levels in the pulmonary artery rings were positively correlated with the degree of Ach-induced relaxation and negatively correlated with the number of pack-years.

**Conclusion:**

SHH pathway genes and proteins are expressed in pulmonary artery rings from smokers, where they modulate endothelial function through VEGF.

## Background

Pulmonary vascular remodelling can occur in smokers, regardless of whether the latter have normal or impaired lung function [1]. These vascular changes may ultimately lead to increased pulmonary vascular resistance and subsequent pulmonary hypertension, which is a negative prognostic factor. Pulmonary endothelial dysfunction is thought to be an early pathophysiological determinant of this vascular remodelling. Endothelial dysfunction has been reported in patients with end-stage chronic obstructive pulmonary disease (COPD) [2], patients with mild COPD, and smokers with normal lung function [3]. The pathophysiological mechanism is complex and still poorly understood but seems to involve an imbalance between vasodilating/anti-proliferative and constrictive/pro-remodelling factors.

Sonic hedgehog (SHH) is a developmental pathway that controls epithelial-mesenchymal interactions during the morphogenesis of various organs (including the lungs) and also influences lung branching [4]. The SHH pathway also plays a key role in cell differentiation and proliferation [5], and in tissue repair after ischaemia [6]. In the absence of SHH, the 12- transmembrane receptor Patched-1 (PTCH1) represses the 7- transmembrane protein Smoothened (SMO), which regulates activation of the GLI family of transcription factors (GLI1, GLI2 and GLI3) [7]. SHH binding to PTCH1 de-represses SMO, which then promotes the formation of the GLI2 transcriptional factor. In the absence of SHH, suppressor of Fused (SUFU) inhibits GLI transcriptional activity. Upon pathway activation by SHH, Fused (STK36) counteracts the suppressor of Fused (SUFU) activity, allowing nuclear translocation of GLI2. The nuclear translocation of this major transcription factor induces the expression of SHH downstream target genes, including GLI1, PTCH1, Hedgehog interacting protein (HHIP, an SHH antagonist) and pro-angiogenic genes (such as the vascular endothelial growth factor (VEGF) and angiopoietins (ANGPT 1 and 2) [8]. The signalling pathway involving GLI is known as the canonical SHH pathway. A number of non-canonical SHH signalling pathways exist, which are independent of GLI transcription. For instance SHH can induce anti-apoptotic effects in endothelial cells and directly modulate endothelial cell phenotype and angiogenic activity (migration and capillary formation) [9–11]. Functional SHH is also expressed in vascular tissues in adults, namely vascular smooth muscle cells, endothelial cells and endothelial progenitor cells [8]. SHH’s beneficial roles in the pathophysiology of the systemic vasculature have recently been elucidated; they include pro-angiogenic, pro-remodelling and proliferative effects on vascular smooth muscle cells [12–16]. Activation of the SHH pathway also induces the release of endothelial NO and corrects endothelial dysfunction following ischaemia-reperfusion [17, 18] and hypertension [19]. Several SHH pathway components (including SHH, SMO, PTCH1 and GLI) are also expressed in human pulmonary arteries, where SHH can induce the proliferation of vascular smooth muscle cells [20].

The observation of low HHIP expression in lung tissue samples from COPD patients has suggested that the disease is associated with changes in SHH signalling [21–23]. Furthermore, tobacco smoking enhanced VEGF expression and the proliferation of vascular smooth muscle cells in the pulmonary artery of smokers (regardless of whether or not the smokers had moderate chronic obstructive lung disease) [1]. VEGF induced NO- and endothelium-dependent relaxation in isolated systemic arteries [24] and bovine pulmonary arteries [25], and VEGF inhibition induced pulmonary hypertension in an animal model [26]. However, the details of SHH signalling in this context are not clear. Hence, the objective of the present study was to establish whether or not the SHH pathway (through its vascular effects and its downstream target VEGF) is involved in pulmonary endothelial dysfunction in smokers.

## Methods

We obtained explants from current smokers, ex-smokers or never-smokers undergoing resection for lung cancer in a university hospital (Hôpital Foch, Suresnes, France) and in the Clinique Val d’Or private clinic (Saint Cloud, France). The study’s objectives and procedures and the use of human lung tissue for in vitro experiments were approved by the local independent ethics committee (*Comité de Protection des Personnes Ile de France VIII*, Boulogne-Billancourt, France). All patients gave their informed consent to the use of the lung tissues for research purposes.

### Tissue preparation

Immediately after excision, lung tissue samples were placed in Krebs-Henseleit solution (mM: NaCl 119, KCl 5.4, CaCl_2_ 2.5_,_ KH_2_PO_4_ 1.2, MgSO_4_ 1.2_,_ NaHCO_3_ 25, glucose 11.7) and immediately transported to our laboratory. After intralobar arteries had been carefully dissected free of parenchyma and adhering connective tissue, several rings (3 to 5 mm in length, and 1.5 to 2 mm in internal diameter) were prepared from a single artery. Some of the rings were used immediately for pharmacological studies, whereas others were snap-frozen and stored in liquid nitrogen for subsequent protein extraction.

To assess endothelial function of pulmonary artery rings isolated from smokers or never-smokers, we evaluated the relaxation produced in response to cumulative, increasing concentrations of acetylcholine (Ach). Under our experimental conditions, endothelial dysfunction was defined as an impaired response to Ach, i.e. a relaxation that was 2 SD below the mean value in never-smokers at an Ach concentration of 10^−4^M (a lack of relaxation or, in some cases, even contraction),.

### Pharmacological experiments

Pulmonary artery rings were mounted in bath organs, as previously described [3]. Briefly, rings were suspended on tissue hooks in 5 ml organ baths containing Krebs-Henseleit solution (pH 7.4) maintained at 37 °C and bubbled with 95% O_2_ and 5% CO_2_. Each preparation was connected to a force displacement transducer (Statham UF-1) and changes in isometric tension were recorded. An initial tension of 1 g was applied to the rings, which were then left to equilibrate for 30 min (with regular changes in fresh Krebs-Henseleit solution) until a stable resting tension (RT1) was obtained. The rings’ responsiveness was confirmed by inducing contraction with KCl (40 mM). The rings were then washed until full relaxation had occurred (resting tension 2, RT2), and were left to rest for 20 min. The rings were then precontracted with L-phenylephrine (PE) dichloride (10^−5^ M), so as to obtain a stable plateau of contraction. Serial dilutions of Ach were then added, in order to establish a cumulative-concentration response curve (10^−10^ to 10^−4^ M). Relaxation induced by Ach was expressed as a percentage of the contraction induced by PE. A contractile response to Ach was expressed as a negative value. Endothelium-independent relaxation was assessed by measuring the response to sodium nitroprusside 10^−5^ M at the end of each experiment.

For each patient, some rings were incubated with various drugs for 45 min after PE precontraction. We used two SHH pathway antagonists (cyclopamine: sc-200929, Santa Cruz Biotechnology, Lexington, KY, USA) and GANT61 (2,2′-[[dihydro-2-(4-pyridinyl)-1,3(2*H*,4*H*)-pyrimidinediyl] *bis*(methylene)]*bis*[*N*,*N* dimethylbenzenamine, Calbiochem, Darmstadt, Germany , ref. 373401)) and an SHH pathway agonist (SAG: 3-chloro-N-[trans-4-(methylamino)cyclohexyl]-N-5[[3-(4-pyridinyl)-phenyl]methyl]-1-benzothiophene-2-carboxamide, sc-212905, Santa Cruz Biotechnology, Lexington, KY, USA). Cyclopamine is a plant-derived alkaloid that binds to the SHH pathway transducer SMO and stabilizes it in an inactive form - thereby blocking SHH signalling [27]. GANT61 inhibits the SHH pathway by specifically blocking the binding of GLI1 and GLI2 to their DNA targets [28, 29]. GANT61 (5 μM) and cyclopamine (0.1 μM) were dissolved in dimethyl sulfoxide (DMSO.

The SHH pathway agonist SAG binds to SMO [27] . SAG was dissolved in water. Certain rings were incubated with recombinant human VEGF 165 (R&D Systems Europe, Abingdon, UK; 1 ng/ml) for 45 min after incubation with PE.

The concentrations of these drugs used in the present pharmacological experiments had previously been determined to be those producing 50% of the maximal effect (i.e. the EC_50_) in pulmonary artery rings (data not shown). All other drugs were purchased from Sigma Aldrich (St Quentin Fallavier, France). All experiments were performed in duplicate. The inter-ring variability was always below 10%.

### RNA isolation and reverse transcriptase – quantitative polymerase chain reaction (RT-qPCR) analysis

Pulmonary artery rings were placed at −80 °C in TRIzol reagent (Invitrogen, Carlsbad, CA) for subsequent mRNA extraction. The RT-qPCR experiments were performed as described in our previous work [30]. Pulmonary artery rings were crushed and homogenized in TRIzol reagent, using a Tissue-Lyser LT ball mill (Qiagen, Courtaboeuf, France). Total RNA was extracted from arterial homogenates using TRIzol. The amount of RNA extracted was estimated by spectrophotometry at 260 nm (Biowave DNA; Biochrom, Cambridge, UK) and the quality of the preparation was assessed in a microfluidic electrophoresis system (RNA Standard Sensitivity kits for Experion, BioRad, Marnes-la-Coquette, France). After treatment with DNase I (Life Technologies, Saint Aubin, France), 1 μg of total RNA was reverse-transcribed (SuperScript III First-Strand SuperMix kit, Life Technologies). The resulting cDNA was then used for RT-qPCR experiments with TaqMan chemistry (Life Technologies). After initial denaturation at 95 °C for 10 min, 20 ng of cDNA were amplified (using Gene Expression Master Mix, Life Technologies) in 40 annealing/extension cycles (95 °C for 15 s and 60 °C for 1 min) in a StepOnePlus thermocycler (Life Technologies). The sample’s fluorescence was measured after each cycle, and the threshold cycle (Ct) of the real-time PCR was defined as the point at which a fluorescence signal corresponding to the amplification of a PCR product was detectable. The reaction volume was 10 μl. The following genes were tested: *SHH*, *PTCH1*, *SMO*, *GLI1*, *GLI2*, *GLI3*, *HHIP1*, *VEGF*, *ANG1*, *ANG2 and SUFU*.

The expression of relevant transcripts in the pulmonary artery rings was analyzed using a specific TaqMan Array based on predesigned reagents (Assay-on Demand, Life Technologies). In order to validate the extraction of intact cellular mRNA and standardize the quantitative data, three reference genes (hypoxanthine phosphoribosyltransferase (*HPRT1*), glyceraldehydes-3-phosphate dehydrogenase (*GAPDH*) and β-glucuronidase (*GUSB*)) were amplified simultaneously.

### Extraction of protein from pulmonary artery rings

At the end of the pharmacological experiments, pulmonary artery rings were frozen in liquid nitrogen for subsequent protein extraction. Total proteins were extracted with a lysis buffer containing NP 40 cell lysis buffer (Invitrogen, Life Technologies), Protease Inhibitor Cocktail for general use, Phosphatase Inhibitor Cocktail 1, Phosphatase Inhibitor Cocktail 2 and PMSF (Sigma-Aldrich). Total proteins were measured with a bicinchoninic acid protein assay kit (Thermo Fisher Scientific, Courtaboeuf, France) on a microplate, according to the manufacturer’s instructions.

### ELISA

The Quantikine ELISA Human VEGF Immunoassay kit (R&D Systems Europe) was used to assay VEGF levels in protein extracts from pulmonary artery rings. VEGF in the samples was captured on microtiter plates precoated with a monoclonal anti-VEGF antibody and detected by an enzyme-linked polyclonal antibody specific for VEGF. The minimum detectable dose of VEGF was below 5.0 pg/mL.

### Statistical analysis

Results are expressed as the mean ± standard error of the mean (SEM). Data were analysed with NCSS9 software (NCSS LLC, Kaysville, UT and GraphPad Prism software (version 5.00 for Windows, GraphPad Software, San Diego, CA). For intergroup comparisons (smokers vs never-smokers), a non-parametric analysis of variance (ANOVA) was followed by Dunn’s test for multiple comparisons. For comparisons of condition (i.e. Ach dose-response curves in the presence and absence of another drug), a repeated-measures ANOVA was followed by a Tukey-Kramer test for multiple comparisons. Fischer’s exact test or the Mann-Whitney test was used to compare categorical variables. To search for a correlation between 2 parameters, a non parametric correlation (Spearman) test was performed, followed by a linear regression. A p value <0.05 was considered to be statistically significant. The quantitative data obtained from RT-qPCR experiments was expressed as the relative expression (2^-ΔCt^), where ΔCt is the difference between the target gene Ct and the mean Ct of the reference genes [31].

## Results

### Subjects

Lung tissues were obtained from 34 current or ex-smokers and compared with a historical series of rings from 8 never-smokers in which Ach-induced relaxation (but not SHH activity or expression) had been characterized. Smoking history was the only demographic or clinical factor that differed significantly when comparing current/ex-smokers with never-smokers (Table 1).

**Table 1:** General characteristics and lung function measurements

Characteristics	Smokers (*n* = 34)	Never smokers (*n* = 8)	*p*
Age, years (mean ± SEM, range)	64 ± 8 [49–87]	56 ± 25 [23–83]	NS
Male/Female ratio	20/14	6/2	NS
Current smokers, *n*	17	NA	NA
Tobacco, pack-years (mean ± SEM, range)	42 ± 25 [10–110]	NA	NA
COPD	4	0	NS
GOLD stage 1, *n*	3	0	NS
GOLD stage 2, *n*	1	0	NS
GOLD stage 3, *n*	0	0	
GOLD stage 4, *n*	0	0	
Prior chemotherapy, *n*	4	1	NS
Hypercholesterolemia, *n*	4	0	NS
Hypertension, *n*	5	1	NS
Diabetes mellitus, *n*	0	0	NS
Treatment with statins, *n*	2	0	NS
Treatment with vasodilators, *n*	4	0	NS

Pack-years: number of cigarette packs smoked per day multiplied by the number of years of smoking

*COPD* chronic obstructive pulmonary disease, defined by post bronchodilator FEV1/FVC < 70% (where FEV1 is the forced expiratory volume in 1 s and FVC is the forced vital capacity), *GOLD* Global Initiative for Chronic Lung Disease - 2011, *NS* not significant, *NA* not appliable

#### Tobacco smoking impairs the relaxation response of pulmonary artery rings

**Fig. 1:**
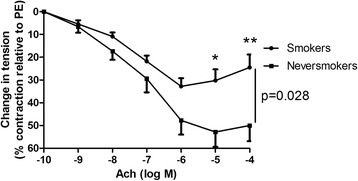
Pulmonary endothelial function, represented as cumulative Ach dose response curves in pulmonary artery rings from smokers (*n* = 34) and never-smokers (*n* = 8). Rings from smokers displayed impaired relaxation in response to Ach, when compared with rings from never-smokers (*p* = 0.028)

The Ach-induced relaxation was much less intense in smokers than in never-smokers (respectively 24 ± 6% vs. 50 ± 7% at Ach 10^−4^M; *p* = 0.028) (Fig. 1).

#### SHH modulation alters pulmonary vasodilation

**Fig. 2:**
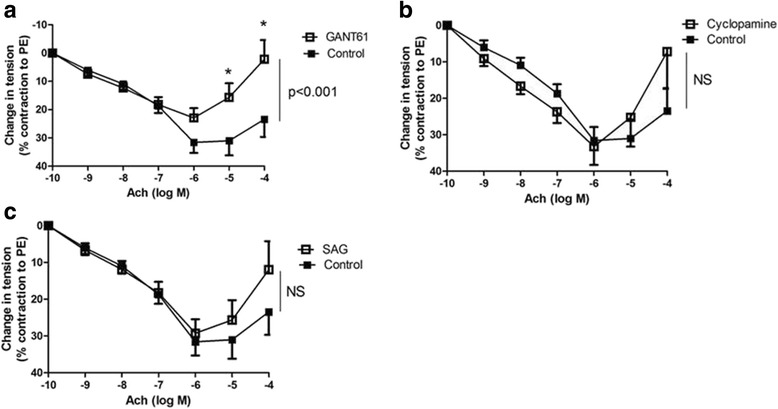
Effect of SHH modulation on pulmonary artery ring relaxation. Treatment with the downstream SHH inhibitor GANT61 altered vasodilation (*n* = 27; *p* < 0.001) (**a**), whereas SHH upstream inhibition by cyclopamine (*n* = 27) had no effect (**b**). SHH activation with SAG (*n* = 27) had no effect (**c**)

We tested the effect of SHH inhibition in pulmonary artery rings from smokers. The downstream SHH inhibitor GANT61 strongly altered vasodilation (2 ± 7% vs. 23 ± 6% at Ach 10^−4^M in the presence and absence of GANT61, respectively; *n* = 27, *p* < 0.001) (Fig. 2a). In contrast, neither upstream SHH inhibition by cyclopamine (*n* = 27; Fig. 2b) nor SHH activation by SAG (*n* = 27; Fig. 2c) had a significant effect on the relaxation response.

#### SHH genes are expressed in pulmonary artery rings

**Fig. 3:**
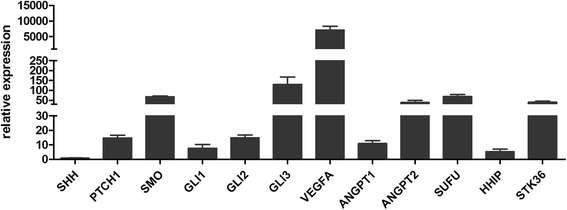
SHH gene expression in pulmonary artery rings. All genes of the SHH pathway are expressed in pulmonary arterial rings from smokers (*n* = 11)

mRNAs from all known genes involved in the response to SHH were expressed in pulmonary artery rings from smokers (*n* = 11; Fig. 3).

#### VEGF restores vasodilation in pulmonary artery rings

**Fig. 4:**
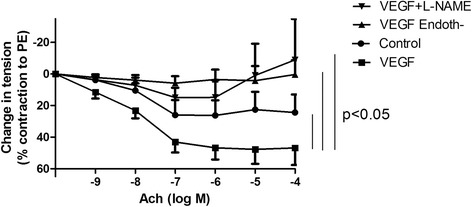
Effect of VEGF on endothelium- and NO-dependant pulmonary relaxation. Treatment with VEGF strongly enhanced the relaxant response to Ach (*n* = 6). This effect was endothelium- and NO- dependent, as shown by the full inhibitory effect of either endothelium removal (Endoth-) or incubation with a NO synthase inhibitor (L-NAME)

To further assess the potential vascular effect of the SHH pathway, we investigated one of its main targets: VEGF. To this end, we tested the effect of recombinant VEGF on the response to Ach in pulmonary artery rings from smokers (*n* = 6). VEGF strongly increased Ach-induced relaxation, and restored the relaxant response to Ach (47 ± 7% vs. 24 ± 7% in the presence and absence of VEGF, respectively; *p* < 0.05) to the level observed in non-smokers. This relaxant response was NO- and endothelium-dependent, as shown by the full inhibitory effect of either endothelium removal or incubation with *N*_ω_-Nitro-L-arginine methyl ester hydrochloride (L-NAME) (Fig. 4).

**Fig. 5:**
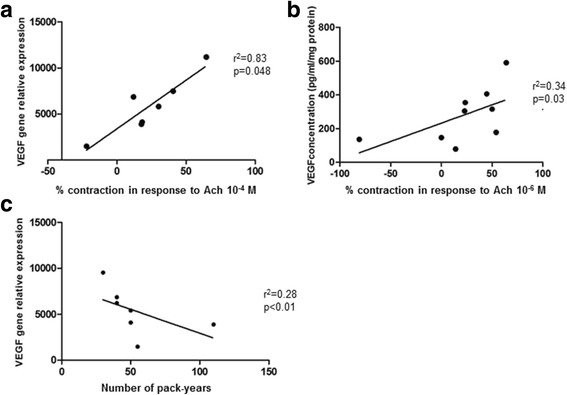
VEGF gene and protein expression levels in pulmonary artery rings, and the effect of tobacco smoking on VEGF gene expression. Levels of VEGF gene (**a**) and protein (**b**) expression in pulmonary artery rings from smokers were correlated with the response to Ach. VEGF gene expression was inversely correlated with the number of pack-years (**c**)

Furthermore, levels of VEGF gene and protein expression in pulmonary artery rings from smokers were correlated with the response to Ach (*n* = 7, r^2^ = 0.83, *p* = 0.048, Fig. 5a, and *n* = 9, r^2^ = 0.34, *p* = 0.03, Fig. 5b, respectively). Lastly, VEGF gene expression in pulmonary artery rings was inversely correlated with the number of pack-years (*n* = 7, r^2^ = 0.28, *p* < 0.01, Fig. 5c).

## Discussion

Taken as a whole, the present results show that the SHH pathway is involved in pulmonary endothelial dysfunction in smokers through the downstream target VEGF. All known SHH pathway genes were found to be expressed in the pulmonary artery rings. The downstream inhibition of SHH by GANT61 reduces pulmonary endothelial–dependent relaxation by downregulating VEGF gene expression. VEGF induces endothelium- and NO-dependent pulmonary relaxation, and VEGF gene and protein levels are correlated with the degree of the relaxation. Incidentally, these results confirmed our previous observation of pulmonary endothelial dysfunction in smokers (probably due to smoking itself) - regardless of the presence or absence of obstructive airway disease [3]. Indeed, there was no difference between the smokers and the never-smokers in terms of clinical characteristics in general and the cardiovascular risk factors usually associated with systemic endothelial dysfunction (such as diabetes or hypertension) in particular. Furthermore, altered relaxant responses were observed in subjects with no obstructive lung disease, and only 4 of the 34 smokers were classified as Global Initiative for Chronic Obstructive Lung Disease (GOLD) stages 1 and 2. Several mechanisms underlie the abnormal relaxation observed in smokers. We recently reported on the role of the ET-1 pathway via enhanced ET-A expression [3]. We have also demonstrated the role of arginase in this dysfunction [32]. SHH is another potential mechanism in vascular dysfunction. The beneficial vascular effects of SHH signalling (especially angiogenesis and neovascularization) were recently described [33]. Several studies have reported on the pro-angiogenic effects of SHH in endothelial cells through canonical or non-canonical pathways [9, 34]. Interestingly, in the context of endothelial function, few studies have shown that SHH carried on microparticles have beneficial effects on endothelial dysfunction in a mouse model through NO release [17, 19]. Our present data provide evidence of a novel role of canonical SHH signalling in tobacco-associated pulmonary endothelial dysfunction. Previous observations of VEGF’s endothelium- and NO-dependent vasorelaxant effects concerned the systemic vasculature and pulmonary arteries from animals [24, 25]. To the best of our knowledge, the present study is the first to show this type of effect in human pulmonary artery samples. VEGF is one of the downstream targets for the canonical SHH pathway. We showed that inhibition of GLI formation by GANT61 alters pulmonary endothelial function. Levels of VEGF gene and protein expression in pulmonary artery rings were correlated with the ring’s degree of relaxation in response to Ach. The expression and role of VEGF in lungs exposed to cigarette smoke are still subject to debate [35, 36]. Interestingly, vascular VEGF gene expression was inversely correlated with tobacco exposure (pack-years) in our patients; this provides further evidence of a harmful effect of tobacco smoke on pulmonary endothelial function via VEGF downregulation.

Interestingly, SHH activation by SAG had no effect on endothelial dysfunction or VEGF expression, nor did SMO inhibition by cyclopamine. There are very few known SHH activators, and all have an effect on SMO or upstream of SMO. The large variety of possible canonical or non-canonical effects that follow SMO derepression (including RhoA/ROCK activation and apoptosis) may account for SAG’s lack of action in our experiments [11, 23, 37]. Downstream activators of SHH are presently lacking.

One of the limitations of our study relates to the fact that we could not assess SHH signalling downregulation in smokers relative to never-smokers (due to the very low frequency of lung resection for cancer in the latter group). We can only state that (i) SHH’s downstream target VEGF has NO- and endothelium-dependant relaxant responses and (ii) VEGF expression is inversely correlated with tobacco smoking load. Another limitation is that the current lack of downstream SHH activators prevented us from testing the effect of downstream SHH activation on pulmonary artery relaxation.

## Conclusion

Our present results evidenced the pulmonary expression of SHH in smokers and suggest that this pathway has beneficial effects on the pulmonary endothelial dysfunction in this setting via the downstream target VEGF. Future research should establish whether (i) this pathway is downregulated in tobacco smokers and (ii) non-canonical pathways are involved in endothelial dysfunction.

## Abbreviations

Ach: Acetylcholine
ANGPT: Angiopoietin
COPD: Chronic obstructive pulmonary disease
FEV1: Forced expiratory flow in 1 second
GOLD: Global Initiative for Obstructive Lung Disease
HHIP: Hedgehog interacting protein
PTCH1: Patched-1
SHH: Sonic hedgehog
SMO: Smoothened
STK36: Fused
SUFU: Suppressor of Fused
VEGF: Vascular endothelial growth factor
